# Sex Differences in Birth Weight and Physical Activity in Japanese Schoolchildren

**DOI:** 10.2188/jea.JE20170078

**Published:** 2018-07-05

**Authors:** Mitsuya Yamakita, Miri Sato, Kohta Suzuki, Daisuke Ando, Zentaro Yamagata

**Affiliations:** 1 College of Liberal Arts and Sciences, Kitasato University, Sagamihara, Kanagawa, Japan; 2 Center for Birth Cohort Studies, Graduate School Department of Interdisciplinary Research, University of Yamanashi, Yamanashi, Japan; 3 Department of Health and Psychosocial Medicine, Aichi Medical University School of Medicine, Aichi, Japan; 4 Division of Human Sciences, Faculty of Education, Graduate School Department of Interdisciplinary Research, University of Yamanashi, Yamanashi, Japan; 5 Department of Health Sciences, Basic Science for Clinical Medicine, Division of Medicine, Graduate School Department of Interdisciplinary Research, University of Yamanashi, Yamanashi, Japan

**Keywords:** birth weight, physical activity, school-aged children

## Abstract

**Background:**

Lower birth weight (BW) is associated with increased chronic disease risk later in life. Previous studies suggest that this may be mediated principally via physical activity (PA). However, the association between BW and PA in children has not been clarified. The purpose of this study was to examine the association between BW and PA in school-aged children in Japan.

**Methods:**

Participants were children from a prospective birth cohort study (Project Koshu) who were born from 1996 through 2002 in rural Japan. BWs were obtained from the Maternal and Child Health Handbook. Data on PA during childhood were collected using a self-reported questionnaire when participants were 9–15 years of age in July 2011. Analysis of covariance was used to evaluate exercise duration; Poisson regression analysis was used to evaluate if the recommended PA amount was met.

**Results:**

Data from 657 children (boys: 54.8%, follow-up rate: 77.6%) were analyzed. Compared with the normal BW group, only girls in the low-BW group had significantly lower PA level (normal BW, 11.4 [standard error, 1.0] hours/week; low BW, 5.8 [standard error, 3.6] hours/week, *P* = 0.010), and were more likely to not meet the recommended PA level (prevalence ratio 1.57; 95% CI, 1.14–2.16).

**Conclusion:**

Low BW was associated with a lower PA level in school-aged girls but not boys. Earlier consideration of BW may be an important public health strategy to prevent physical inactivity in school-aged girls.

## INTRODUCTION

Physical activity (PA) is one of the most important contributors to maintaining optimal health, and considerable evidence suggests that sufficient PA has the potential to prevent numerous diseases and provide health benefits to people of all ages.^1^^–^^4^ Studies suggest that school-age PA influences adult PA and health status in later life.^5^^–^^8^ PA promotion may be effective if initiated in childhood or earlier in life.

However, globally, 81% of school-going children and adolescents aged 11–17 years do not meet the recommended guideline^9^ of at least 60 minutes of moderate-to-vigorous PA daily.^1^ Among Japanese children, no representative data is available on whether PA levels are being met according to the official national PA guidelines.^10^^,^^11^ According to the Japan sports agency survey, which assessed adequacy of PA based on other criteria (420 min/week), 44.3% of boys and 67.3% of girls in 5th grade (aged 10–11 years) and 15.8% of boys and 39.5% of girls in the second year of junior high (aged 13–14 years) engaged in exercise for less than 420 min per week, not including that in physical education classes.^12^ Hence, the majority of Japanese school-aged children do not achieve PA recommendations.

Therefore, to develop effective public strategies promoting PA in children and adolescents, a better understanding of its intrinsic and extrinsic determinants is required. To date, a broad range of factors has been investigated, including interpersonal, demographic, biological, psychological, social, cultural, and environmental factors.^13^^–^^16^ In addition, recently, it has been suggested that birth weight (BW) is associated with PA later in life.^17^^–^^19^ Numerous epidemiological studies substantiated a close association between low BW and an increased risk of chronic diseases.^20^^–^^22^ A meta-analysis suggested a lower probability of undertaking leisure-time PA in adolescent and adults with a low or high BW,^23^ which indicated that the association between BW and higher risk of metabolic diseases in adulthood could partly be explained by lower rates of PA in childhood.^19^^,^^23^ Meanwhile, not all studies have consistently confirmed the association between BW and PA among children and adolescents.^24^ Moreover, to the best of our knowledge, no studies have investigated this association in Japanese school-aged children.

The aim of this study was to examine the association between BW and PA in school-aged children in Japan.

## METHODS

### Study participants

The study participants comprised children born in the Enzan area of Koshu City, Yamanashi Prefecture, Japan between April 2, 1996 and April 1, 2002. The participants were from Project Koshu, a community-based prospective birth cohort study. Project Koshu is an ongoing study started in 1988, in which all expectant mothers who responded to a survey during the obligatory visit at the city office for pregnancy registration were recruited into the cohort. The children were followed from birth onwards. Further details of the project have been reported elsewhere.^25^ The data of the present study were based on a follow-up study carried out during 2011.

This study was approved by the Ethics Review Board of the University of Yamanashi School of Medicine and conducted in accordance with the Guidelines Concerning Epidemiological Research, with cooperation of Health Promotion Division and Board of Education of the Koshu City administration office. Informed consent was obtained from the participants.

### Measurements

#### Assessment of BW

Data regarding the sex of the infants, BW and birth length, and gestational age at delivery were obtained from the data recorded in the Maternal and Child Health Handbook by the obstetrician or midwife in charge of delivery. This handbook is an official publication containing guidelines for obstetric professionals and pregnant women. These data were based on birth registration. Low BW was defined as BW <2,500 g. As a previous study provides evidence that low (<2,500 g) and high (≥3,500 g) BWs are associated with a lower probability of undertaking leisure-time PA,^23^ BW was categorized into the following three groups: low (<2,500 g), normal (2,500–3,499 g), or high (≥3,500 g).

#### Assessment of PA

Data concerning PA was obtained from children using a self-reporting questionnaire conducted in July 2011. The following question was used to obtain PA levels: “How many hours per week do you usually spend on PA except for physical education class (for example, school club activity, sports club activity, or swimming or tennis school, etc)?” This simple question has been used for other studies in children and has shown acceptable validity.^26^ To investigate whether there is a difference between BW and PA in children who did and did not meet the guideline for PA for Japanese children,^11^ participants were classified into two groups according to whether they met the recommended guidelines (≥60 min/day; ie, ≥7 hours/week).

#### Assessment of covariates

Gestational age, age in months, body mass index (BMI) during childhood, and parental educational levels were identified as potential confounders based on previous studies.^18^^,^^27^^–^^29^ Age and BMI of children were collected via physical measurements taken during medical checkups conducted at elementary and junior high schools, which are measured annually in April for each grade, in accordance with Japanese School Health and Safety Law. BMI (kg/m^2^) was calculated from height and weight. Participants were classified as overweight (equivalent to BMI ≥25 kg/m^2^ at 18 years old) or non-overweight based on age- and sex-specific international cut-off points for BMI.^30^ The highest parental educational levels were collected from mothers using a self-reported questionnaire at pregnancy registration. The responses were collapsed into two categories by number of education years: ≤12 years (up to high school) and ≥13 years (college or higher). A variable was then created combining the highest education level by each parent as follows: ≤12 years (both parents), ≥13 years (only father), ≥13 years (only mother), and ≥13 years (both parents).

### Statistical analysis

Data were analyzed separately for boys and girls based on the results of a previous study that revealed sex difference in PA levels.^31^ Analysis of covariance was used to compare the mean PA time among BW groups. When prevalence of an outcome is common in the study population (>10%), the odds ratio derived from logistic regression tends to overestimate the strength of an association.^32^^,^^33^ The prevalence of children not meeting the recommended guideline for PA was high. Therefore, Poisson regression analyses with robust variance estimators were conducted to examine the associations between BW and the proportion not meeting recommended PA, and prevalence ratio (PR) was used instead of odds ratio. Then, the analyses were adjusted for gestational age, age in months, age- and sex-specific BMI categories (overweight or non-overweight), and parental education levels.

All statistical analyses were performed using SPSS statistical software version 19.0 (SPSS Inc., Chicago, IL, USA). A *P*-value <0.05 (two-sided) was considered statistically significant.

## RESULTS

### Characteristics of participants

During the study period, maternal information during pregnancy and BW were collected from 847 children. Of these, the complete follow-up data in 2011 were collected from 657 children (boys: 54.8%). Therefore, the follow-up rate at 9–15 years of age was 77.6%. Table 1 shows the characteristics of the participants. The range of BW, birth length, and gestational age was 1,404–4,336 g, 33–58 cm, and 33–42 weeks, respectively.

**Table 1.** Characteristics of study participants

	Boys (*n* = 360)		Girls (*n* = 297)		*P*-value^a^
**Birth**					
Birth weight					
g, mean (SD)	3059.0	(386.4)	3015.7	(387.0)	0.153
Low birth weight (<2,500 g), *n* (%)	27	(7.5)	22	(7.4)	0.960
Normal birth weight (2,500–3,499 g), *n* (%)	293	(81.4)	244	(82.2)	
High birth weight (≥3,500 g), *n* (%)	40	(11.1)	31	(10.4)	
Birth length, cm, mean (SD)	49.0	(2.1)	48.8	(2.1)	0.197
Gestational age, weeks, mean (SD)	38.8	(1.4)	39.1	(1.4)	0.004
Parental education, *n* (%)					
≤12 years (both parents)	119	(33.1)	100	(33.7)	0.358
≥13 years (only father)	49	(13.6)	33	(11.1)	
≥13 years (only mother)	67	(18.6)	45	(15.2)	
≥13 years (both parents)	125	(34.7)	119	(40.1)	

**Childhood**					
Age, years, mean (SD)	12.0	(1.7)	12.1	(1.9)	0.740
Height, cm, mean (SD)	149.6	(13.0)	147.8	(10.1)	0.050
Weight, kg, mean (SD)	42.1	(12.1)	41.7	(10.7)	0.693
BMI, kg/m^2^, mean (SD)	18.4	(3.0)	18.8	(3.2)	0.118
BMI category, *n* (%)					
Non-overweight	313	(86.9)	263	(88.6)	0.533
Overweight	47	(13.1)	34	(11.4)	
Physical activity					
hours/week, mean (SD)	13.5	(8.6)	10.8	(9.3)	<0.001
Less than recommended (<7 hours/week), *n* (%)	91	(25.3)	133	(44.8)	<0.001

BMI, body mass index; SD, standard deviation.

Data are means (SD) or number (percentage).

^a^The *t* test for continuous and the χ^2^ test for categorical variables.

There was no significant difference in birth data between boys and girls, except for gestational age. Height and PA time of boys were significantly higher than those of girls; thus, girls were more likely not to meet the recommended PA (Table 1).

### Differences in PA time according to BW categories

**Figure 1.**
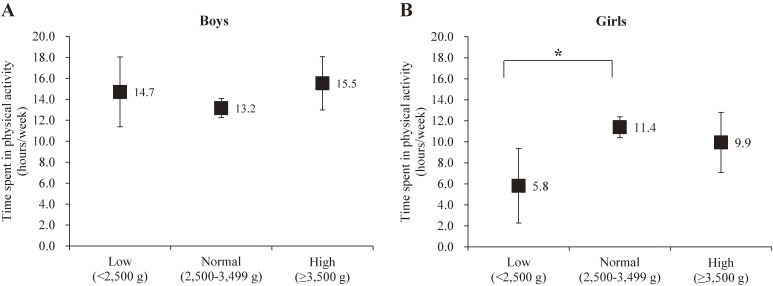
Association between birth weight categories and physical activity time in boys (A) and girls (B). Each plot is presented as estimated marginal means (standard error). Physical activity time was adjusted for age in months, age- and sex-specific body mass index category (overweight or non-overweight), gestational age, and parental education level using analysis of covariance. ^*^ indicates the significance of Low versus Normal (mean difference, −5.57; 95% confidence interval, −10.1 to −1.04, *P* = 0.010; Cohen’s effect size (d) = 0.68).

No significant differences were found in PA time among BW categories in boys (Figure 1A). There was a significant difference in PA time spent between the low-BW and normal-BW groups in girls (Figure 1B). No significant differences were observed between normal- and high-BW groups and high- and low-BW groups in girls.

### BW and attainment of recommended PA

In girls, when compared with the normal-BW group, those in the low-BW group were more likely not to meet the recommended PA (Table 2). This association remained when adjusted for all confounders. For the high-BW group, no significant association was observed. No significant associations were found for boys.

**Table 2.** Association between birth weight categories and not meeting the recommended physical activity at aged 9–15 years

Birth weight categories	Physical activity (<7 hours/week), %	Crude PR	(95% CI)	Adjusted PR^a^	(95% CI)
Boys					
Low (<2,500 g)	25.9	0.96	(0.50–1.87)	0.83	(0.43–1.61)
Normal (2,500–3,499 g)	27.0	1.00	(reference)	1.00	(reference)
High (≥3,500 g)	12.5	0.46	(0.20–1.08)	0.50	(0.22–1.16)

Girls					
Low (<2,500 g)	68.2	1.57	(1.14–2.16)	1.59	(1.12–2.26)
Normal (2,500–3,499 g)	43.4	1.00	(reference)	1.00	(reference)
High (≥3,500 g)	38.7	0.89	(0.56–1.42)	0.96	(0.65–1.43)

CI, confidence interval; PR, prevalence ratio.

^a^Adjusted for age in months, age- and sex-specific body mass index category (overweight or non-overweight), gestational age, and parental education level.

## DISCUSSION

This study examined whether BW was associated with PA in children aged 9–15 years in Japan. The results demonstrated that girls with low BW have a significantly lower activity level compared to girls with normal BW, with a medium-to-large effect size. In addition, when the hours per week were converted to minutes per day (min/day), the mean time spent in physical activity was 97.7 min/day for the normal-BW group and 49.9 min/day for the low-BW group. These results indicate that the normal-BW group met the recommended PA level, but the low-BW group did not. Meanwhile, this significant difference was not observed in boys.

Several studies have investigated the association between BW and PA in children and adolescents.^24^^,^^34^ Gopinath et al^18^ showed that children aged 12 years with low BW participate in less outdoor PA. Although the exact mechanisms for the difference in time spent in PA between low- and normal-BW individuals are unknown, previous studies have shown that low BW could be related to reduced physical capacity, including reduced muscle strength^35^ and insufficient anaerobic capacity.^36^ These negative physiological factors could reduce the willingness to participate in competitive PA because of early fatigue and a reduced ability to perform PA.^18^^,^^23^ However, our study found this association only in girls. It is unclear why this sex difference was observed; we speculate that it may be explained by the difference in time spent in sports activities and PA between boys and girls. In Japan, available opportunities for girls to participate in sports or PA are limited compared to boys. Parents spend less money on sports activities and PA for girls than boys, whereas they spend three times more on artistic activities for girls.^37^ Therefore, girls with low BW who are thought to have poor exercise capacity may have been participating in non-exercise activities, such as music and the arts (eg, piano lessons). In addition, animal studies have indicated that intrauterine growth restriction leads to low BW and causes decreased PA, especially in female mouse offspring.^38^ Although it is unclear why this occurred, the mechanism of sex-specific alterations in epigenetic regulation in the hypothalamus and other regions of the central nervous system has received attention^39^; it has been suggested that androgen-mediated masculinization of the male mouse brain (which occurs during late fetal development) protects the central nervous system against the deleterious effects of fetal growth restriction.^40^ It is uncertain whether similar processes occur in humans; thus, extensive future studies are required to elucidate the molecular mechanism by which fetal growth restriction may lead to a lack of PA participation.

Meanwhile, other studies that objectively measured PA with an accelerometer did not confirm the association between BW and PA in children and adolescents.^24^^,^^34^ It is possible that objective measures of PA and self-reported PA are capturing different aspects of PA. A previous study suggested that self-reported PA may capture only specific types of exercise and leisure-time PA, whereas accelerometers measure all body movement throughout a measurement period.^41^ Although sex was adjusted for in all of these studies, PA levels between boys and girls is markedly different.^31^^,^^42^ Additionally, PA frequency and intensity among school-aged children across domains (transport, school, leisure and organized sports activities, and home) is markedly different.^43^^,^^44^ Thus, future studies require stratification by sex and PA domain.

In a meta-analysis including Scandinavian adolescents and adults, both low and high BW were inversely associated with self-reported leisure PA.^23^ However, our study did not observe an association between high BW and PA time. This may be because of the difference in range of BW. Although BW of the previous study exceeded 4,500 g or 5,000 g, in our study, the highest was 4,300 g. In addition, the biological mechanism explaining the relationship between high BW and physical inactivity could be related to insufficient aerobic capacity or reduced motivation to engage in PA.^44^ Therefore, children of high BW would be more likely to be obese in adulthood through increased physical inactivity. Moreover, the associations between BW and chronic disease that are based on obesity tend to be more apparent later in life.^21^^,^^22^ Similarly, the association between high BW and PA may also tend to be more pronounced in adulthood.

The strengths of this study include its prospective cohort design and relatively high follow-up rate. In addition, the birth parameters ensured validity because they were obtained from the Maternal and Child Health Handbook. However, our study had the following limitations. First, children with low BW were relatively small in number because only one rural area in Japan was used for the study, resulting in a small sample. Future studies with different populations and more participants are required. Second, PA levels were self-reported. However, the simple question has reasonable agreement with the accelerometer, and a single-item measure was found to be equally valid and reliable as an in-depth questionnaire.^45^ Further studies using a combined assessment of subjective and objective methods are needed to evaluate PA more accurately. Finally, although we adjusted for several important confounders, we could not exclude unmeasured confounding variables, such as genetic factors and dietary information.

In conclusion, this study found that Japanese school-aged girls, but not boys, with low BW were less likely to spend time in PA at aged 9–15 years old. Although further prospective studies are needed, our study suggests that low BW may be an early predictor of physical inactivity in childhood. Therefore, parents of low-BW children should encourage them to be active and to participate in noncompetitive PAs. Additionally, to prevent physical inactivity in childhood, especially for girls, an important public health strategy should be implemented that is aimed at encouraging participation in PA for low-BW children. These may lead to prevention of chronic disease in adulthood.
